# Incremental value of cardiac magnetic resonance in the characterization of patients referred for left ventricular non compaction evaluation

**DOI:** 10.1186/1532-429X-13-S1-P287

**Published:** 2011-02-02

**Authors:** Chiara Lanzillo, Mauro Di Roma, Monia Minati, Luciano Maresca, Luigi Sciarra, Francesca Nuccio, Lucia Di Napoli, Leonardo Calò, Ernesto Lioy, Paolo Preziosi

**Affiliations:** 1 Policlinico Casilino, Rome, Italy

## Introduction

Left ventricular non compaction (LVNC) is a rare cardiomyopathy due to a disorder of myocardial morphogenesis as the result of interruption during embryogenesis of the normal myocardium compaction. However, limited information is available regarding the actual impact of cardiovascular magnetic resonance (CMR) findings in reaching a diagnosis of LVNC in a population of patients(pt) referred to a CMR laboratory.

## Purpose

Aim of the study was to evaluate the incremental value of CMR over preliminary clinical/instrumental data in characterizing a group of consecutive pt referred for suspected LVNC.

## Methods

From October 2008 to September 2010, a total of 14 consecutive pt(10 males; mean age 32 ± 15 years, range 15-49 years) underwent CMR (1.5 T, Intera, Philips) to exclude LVNC. All pt presenting a LVNC suspected on echocardiography criteria (absence of any coexisting cardiac anomalies, prominent trabeculation and deep intertrabecular recesses in short axis view end systolic images, NC/C > 2 or with more than three prominent trabeculations, Intertrabecular spaces filled by direct blood flow from the ventricular cavity on color Doppler imaging) were included and underwent CMR. A segment was regarded as non-compacted if the visual appearance clearly suggested the presence of two myocardial layers with different degrees of tissue compaction. The segment with the most pronounced trabeculations was chosen for measurement of the thickness of the non-compacted(NC) and the compacted myocardium(C) perpendicular to the compacted myocardium. The NC/C ratio in diastole was calculated and the maximal ratio was then used for analysis. LVNC on CMR cine sequences was defined as a diastolic ratio of NC/C > 2.3.

## Results

LVNC was ruled out in 10 pt, inclusion of CMR data allowed to reach a diagnosis for LVNC in 4 (28%) patients. Diagnosis other than LVNC were obtained in 3 patients, including: acute or chronic myo-pericarditis (n = 2); dilated cardiomyopathy (n = 1).

Conclusions:In a population of patients with suspected LVNC, the proportion of cases with a confirmed diagnosis of LVNC is significantly impacted by CMR, proving the high diagnostic accuracy of CMR and allowing the detection of previously unrecognized conditions other than LVNC.

